# Modulating the Interplay Between Impulsivity and Interoception Through HD-tDCS to the Right Insula and Anterior Cingulate Cortex

**DOI:** 10.3390/biomedicines14030519

**Published:** 2026-02-26

**Authors:** Riccardo Pirone, Irene Gorrino, Anna Vedani, Carlotta Maiocchi, Giulia Mattavelli

**Affiliations:** 1 IUSS Cognitive Neuroscience (ICoN) Center, Scuola Universitaria Superiore IUSS, 27100 Pavia, Italy; riccardo.pirone@iusspavia.it (R.P.); irene.gorrino@iusspavia.it (I.G.); anna.vedani@iusspavia.it (A.V.); giulia.mattavelli@iusspavia.it (G.M.); 2 Cognitive Neuroscience Laboratory of Pavia Institute, Istituti Clinici Scientifici Maugeri IRCCS, 27100 Pavia, Italy

**Keywords:** HD-tDCS, interoception, cognitive impulsivity, insula, cingulate cortex, interoceptive-cognitive integration

## Abstract

**Background:** Interoception has been proposed as a key mechanism underlying impulsive behaviours, including maladaptive eating. However, the brain mechanisms supporting the interaction between interoception and impulsivity across different reward types remain unclear. This study investigated whether modulating the right insula and the dorsal anterior cingulate cortex (dACC) using high-definition transcranial direct current stimulation (HD-tDCS) could affect interoceptive accuracy and impulsive decision-making. **Methods:** Model-based HD-tDCS montages were defined to target the right insula and dACC. Two behavioural paradigms were administered: (i) the heartbeat detection task (HBD) to assess interoceptive accuracy and (ii) two versions of the delay discounting (DD) task with food and monetary rewards to measure impulsivity. Heart rate variability (HRV) was recorded as an index of autonomic activity. HD-tDCS was delivered online during the HBD, while DD tasks were completed offline. Twenty-four participants took part in four sessions in a within-subject design: baseline DD tasks, anodal HD-tDCS targeting the insula, dACC, or sham stimulation. **Results:** Stimulation of both the insula and dACC reduced participants’ ability to detect synchronous heartbeat while improving accuracy in exteroceptive trials. Discounting rates significantly increased following insula stimulation. Moreover, HD-tDCS effects on DD performance varied depending on reward type. **Conclusions:** These findings suggest differential contributions of the dACC and insula in interoceptive and exteroceptive processing and support the effect of HD-tDCS combined with interoceptive tasks to modulate impulsive decision-making. Reward-specific effects highlight the importance of stimulus type when designing interventions for impulsive eating behaviours.

## 1. Introduction

The interplay between physiological body signals and decision-making has been a central topic in cognitive neuroscience since Damasio proposed the somatic markers hypothesis more than 30 years ago [1,2]. Damasio and colleagues’ seminal studies revealed riskier choices in individuals with lower physiological arousal reactions while performing gambling tasks [3] and attributed a crucial role to the integration of signals from the internal body in decision-making processes. Since then, researchers have investigated the association between physiological signals of body activations and decision-making using different methodologies, as well as the role of the ability to perceive those signals (i.e., interoception) in modulating risky behaviour and impulsivity [4,5]. Better interoceptive capacity has been reported in individuals with lower levels of impulsivity and risk-taking behaviour, or greater aversion to losses, and in market traders with better financial outcomes, suggesting that interoception could contribute to more effective decision-making [6,7,8,9]. In addition, interoceptive accuracy has been characterised as an important health-promoting skill due to its association with emotion regulation and mental well-being [10,11,12]. Besides this, heart rate variability (HRV), a marker of vagal activity, has been associated with adaptive autonomic nervous system (ANS) functioning, which in turn supports cognitive flexibility, attentional resources, and improved emotion regulation [13,14].

The combined influence of the perception of bodily signals and impulsivity is further called into play when decisions concern food and eating. On the one hand, it is documented that interoceptive capacity contributes to healthy eating, with data showing reduced interoceptive accuracy in individuals with obesity, and altered interoception at both behavioural and brain levels in patients with eating disorders (EDs) [15,16,17]. On the other hand, dysregulated eating behaviour has been related to high impulsivity, impaired inhibitory control, and altered neural responses to reward in decision-making [18,19,20]. Despite this evidence, a crucial issue in nutritional neuroscience research concerns the impact of different reward stimuli (i.e., money/food) in driving decision-making and eating behaviour. Some previous studies using the delay discounting paradigm (DD), which measures the preference for smaller immediate versus larger delayed rewards [21,22], have reported a consistent association between impulsivity in DD and weight status, both with food and monetary stimuli. However, evidence to drive a conclusion on the differences between the two stimuli appeared insufficient [23]. At the brain level, monetary and food reward stimuli recruit partially overlapping networks in the striatum, prefrontal and insular cortices, even though some regions showed reward-type specific activation [24] and were influenced by weight status or homeostatic condition. This suggests that the network plays a role in integrating interoceptive and exteroceptive information (i.e., increased responsivity to food when hungry) and in driving behaviour based on these signals [25,26]. In particular, consistent data showed stronger activations for food compared to other rewarding stimuli in the insular cortex [11,24], whose responses correlated with body mass index (BMI) during inhibitory control [27], and have been related to decision-making processes with food stimuli [28]. Thus, converging evidence from different experimental paradigms highlighted the crucial role of the insula in the network tracking risk decision-making behaviour, reward value of food, and interoceptive awareness, suggesting its importance in the dysfunctional eating behaviours, likely related to its regulatory function between interoceptive and reward-based decision-making processes [17,29,30,31]. Another crucial cortical hub for motivated behaviour is the dorsal anterior cingulate cortex (dACC), which is functionally connected with the insula and together form the anterior cingulate–insular network. This circuit enables the integration of interoceptive information with the reward value and emotional salience of the stimuli to allocate control resources and regulate behaviour [32,33]. Notably, the dACC is involved in the executive control network recruited for performing the DD task, in particular when high-conflict options are presented [21], and altered dACC responses during this paradigm have been reported in patients with EDs [34,35]. Specifically in the context of food stimuli and eating behaviour, it has also been shown that dACC activity during food regulation predicted craving and food consumption over time [36], while its connectivity with mesolimbic regions and supramarginal gyrus was modulated by metabolic state during reward decision-making, supporting the role of the dACC in regulating behaviour according to the salience of food stimuli [26].

Interestingly, the insula and dACC have been identified as common core regions of structural and functional alterations in several psychiatric conditions, which emphasises their crucial role in adaptive behaviour and the translational implications of understanding their functional mechanisms to promote innovative neuromodulation protocols with therapeutic purposes [37,38]. Both regions are located deep within the cerebral cortex, the insula in the lateral sulcus and the dACC in the interhemispheric fissure, challenging the application of non-invasive brain stimulation protocols. One possibility is to apply transcranial magnetic stimulation (TMS) with an H-coil, although this method is associated with a certain level of discomfort for patients [39,40], and data on the efficacy in modulating the insula and dACC are partially inconsistent [41,42,43,44]. Another promising tool is the combination of transcranial direct current stimulation (tDCS) and optimised modelling of current flow in the brain, using multiple electrode configurations to apply high-definition (HD)-tDCS [45,46]. However, despite the growing body of literature on HD-tDCS, findings on insula and dACC stimulation remain preliminary. Previous tDCS studies targeting the insula have applied different montages and provided conflicting results. Modulations of interoceptive capacity [47], HRV, and positive affect [48,49] have been reported by studies using bipolar montages over fronto-temporal sites, with the current flow likely reaching the insula, but these effects could not be clearly disentangled from those of the surrounding areas due to current diffusion using large electrode patches [50]. Instead, two studies targeting the insula with HD-tDCS reported null results regarding effects on (i) interoceptive and emotional processing [51], and (ii) decision-making and executive control [52]. Similarly, contrasting results are available for HD-tDCS of dACC: while some studies reported the absence of significant behavioural modulation on cognitive/emotional interference [53] or inhibitory control [54], other studies found that anodal and cathodal stimulation influenced cognitive and emotional interference in a Stroop task, respectively [55], and increased conflict resolution and both loss and risk aversion in gambling tasks [56]. In summary, the available literature demonstrates the role of the insula and dACC in integrating interoceptive and reward processes, but it also emphasises the importance of a better understanding of the mechanisms underpinning these functions and of the effectiveness of neuromodulation protocols targeting the two regions. To the best of our knowledge, there are no previous studies applying comparable HD-tDCS protocols targeting the insula and dACC to assess the effects on interoception and impulsivity in decision-making with different rewards. To address this issue and further explore the interplay between interoception and impulsivity, the present study adopted a single-blind, within-subjects design. Three model-based anodal HD-tDCS sessions were applied online during an interoceptive task, targeting the right insula, dACC, or in sham mode. Impulsivity was measured offline using a DD paradigm with food and money stimuli.

In particular, the heartbeat detection (HBD) task was used as an index of interoceptive accuracy based on previously developed paradigms [29]. This task required participants to detect whether a tone triggered by the R-spike in their electrocardiogram (ECG) was synchronous or asynchronous with their own heartbeat. In control trials, assessing exteroceptive attention, participants were asked to detect whether a sequence of tones was identical or contained a different note [57]. In three separate sessions, counterbalanced across participants, the heartbeat detection task was completed by participants while they were submitted to 20 min of real/sham HD-tDCS to the right insula or dACC. HRV was also measured as a physiological index of neurocardiac function to measure heart–brain interactions and dynamic non-linear ANS process during the task [58]. Indeed, there is evidence suggesting a relationship between HRV and interoception, although few studies have directly combined the two measures with partially inconsistent results [14,59,60,61]. Finally, to assess impulsivity with food and monetary reward stimuli, comparable intertemporal choice tasks were developed to estimate individual levels of DD based on well-established psychophysical models [62]. The DD tasks were completed at baseline and following the three HD-tDCS sessions.

This design allowed us to evaluate the potential modulatory effect of increasing interoceptive attention during the heartbeat detection task on impulsive decision-making, possibly boosting the activity of the anterior cingulate–insular network by means of HD-tDCS applied to either of the two cortical regions during the interoceptive task. Our main hypotheses were: (1) to detect an effect of insula stimulation on the heartbeat detection task, (2) to modulate impulsivity when DD was performed following HD-tDCS sessions applied online with the interoceptive task, (3) to find an increased neuromodulatory effect following real HD-tDCS sessions, particularly for food stimuli, and finally (4) to find a relationship between HRV, interoceptive accuracy and impulsivity.

## 2. Materials and Methods

### 2.1. Participants

Twenty-four healthy right-handed university students (12 males and 12 females; mean age = 25.5 and s.d. = 2.28) participated in the study, which was approved by the local Ethics Committee. Sample size was estimated based on a previous study stimulating the insula and assessing the effect of tDCS condition on interoceptive accuracy [47], reporting an effect size of partial η2 = 0.22, leading to an estimated sample size of 20 participants assessed in three separate sessions (α = 0.05; 1 − β = 0.8). Participants were screened with an ad hoc questionnaire assessing eligibility for non-invasive neurostimulation protocols [63] and the presence of the following exclusion criteria: history of, or susceptibility to migraine and seizures; presence or history of neurological or psychiatric disorders, substance abuse or dependence, brain surgery, tumour or intracranial metal implantation; current use of psychoactive medications, pregnancy; presence of pacemaker or other implanted devices. Safety guidelines for the application of tDCS were followed [64], and all participants signed informed consent forms before starting any experimental procedure.

### 2.2. Questionnaires

Participants completed a set of questionnaires, administered via an online survey platform (Limesurvey 3.28.77) [65], before the experimental procedure to assess manual dominance (Edinburgh Handedness Inventory (EHI); [66]) and to evaluate interoceptive and emotion regulation capacity in daily life. The Body Perception Questionnaire-22 (BPQ-22; [67,68]) assessed body awareness and the responsiveness of the autonomic nervous system (supradiaphragmatic and subdiaphragmatic reactivity). The Dutch Eating Behaviour Questionnaire (DEBQ; [69,70]) evaluated different eating styles (i.e., emotional, external, and restrained eating). The Difficulties in Emotion Regulation Scale-Short Form (DERS-SF; [71,72]) measured different dimensions of emotion regulation (impulse control, emotional awareness, acceptance of emotion, and emotion regulation strategies). The Multidimensional Assessment of Interoceptive Awareness-Version 2 (MAIA-2; [73]) measured interoceptive awareness (i.e., trust and attention regulation towards one’s bodily sensations). The Psyflex [74] assessed different facets of psychological flexibility within a limited time frame (i.e., 7 days).

### 2.3. Heartbeat Detection Task and ECG Recording

The heartbeat detection task was implemented in E-prime software (Version 3.0.3.80) [75] based on methodological recommendations from previous studies [29,57]. Participants were seated comfortably in front of a screen in a shielded, silent cabin, and a BIOPAC MP36 amplifier system (1000 Hz: BIOPAC Systems, Inc., Goleta, CA, USA) running AcqKnowledge 5.0.10 was used to record participants’ ECG with a standard lead II configuration. The R-waves were computed adaptively and generated a 5-V trigger when the signal reached 80% of the R-peak’s height, so that the peak triggered a sound via a trigger box (EMS Trigger Box 1.04-GeMSTR17006; EMS s.r.l., Bologna, Italy) embedded in the E-prime configuration, which was delivered through two speakers positioned next to the screen. The task consisted of 40 trials asking participants to decide whether a series of tones were synchronous or asynchronous to their own heartbeat (Heart trials), or whether all the tones in the series were identical or one was different (Note trials). At the beginning of each trial, either the word “Heart” or “Note” appeared on the screen to instruct participants on the type of trial. Then, 12 tones were presented, followed by the forced-choice option Synchronous/Asynchronous or Same/Different, respectively. Participants responded by pressing two buttons (“B” = left; “N” = right) on the computer board using the middle and index finger of their right hand. Sounds were 800 Hz and 1000 Hz tones lasting 100 ms, which were delivered at the R-waves peak or following a 700 ms delay. Half of the trials presented Synchronous tones and half of the trials presented the same tones; thus, 8 configurations were presented reflecting permutations of Heart/Note type of trials, tones Synchronous/Asynchronous to individual heartbeat, and Same/Different tones within the series. The total task duration was approximately 13 min.

While participants completed the HBD task, ECG signals were continuously recorded, and task triggers were embedded in the trace to enable the examination of task-specific links between interoceptive performance and physiological changes.

### 2.4. Delay Discounting Task: Stimuli and Procedure

This decision-making task required participants to choose between a reward delivered immediately (“now trials”) or earlier (“not now” trials) than an alternative option consisting of a larger reward delivered after a variable delay relative to the sooner option. The amounts of the two rewards, the time of delivery for the sooner and later options, as well as the distance between them, varied systematically across trials. In each session, participants completed two blocks of 80 trials, in which they were instructed to respond by considering that the rewards referred to money or food. To take into account potential individual differences in preferences for sweet or savoury foods, participants selected their preferred food, between chocolate and chips, before starting the experimental sessions (i.e., before performing the baseline). Then the instructions clarified that the number displayed in each trial of the task referred to either monetary units or the number of chocolates/chips. The 80 trials were created by varying (i) the timing of the sooner option (today/two weeks), (ii) the later option delay (two/four weeks after the sooner option), and (iii) the relative difference between the sooner and later options in reward amount (the later option was 0.5, 1, 5, 10, 15, 20, 25, 30, 50, or 75% larger than the sooner option). Following Figner et al. [76], to avoid identical trials, the reward amount of the sooner option was pseudo-randomly drawn from a normal distribution with a mean of 45 (economic units) and 15/85 as minimum/maximum. Sets of 40 trials were created by the full factorial combination of these variables and repeated twice to obtain 80 trials per block. Therefore, eight sets of equivalent but non-identical stimuli were used for money/food blocks in four sessions of the task (baseline, HD-tDCS to the insula, HD-tDCS to the dACC, and sham HD-tDCS). The task was administered using the E-prime software (Version 3.0.3.80 [75]). Each trial began with a fixation cross (1000 ms), followed by the presentation of the sooner (left side) and later (right side) options, which remained on the screen until response or for a maximum of 3000 ms. Participants responded by pressing two buttons corresponding to the left and right options on the screen (“B” = left, “N” = right). Each block started with a recall of the task instructions and an image indicating the type of reward. Trials were presented in randomised order within each block, which was further divided into two subsets of 40 trials to allow participants a break of 2500 ms, during which the image of money/food appeared on the screen to recall the type of reward. Total task duration was approximately 8 min.

### 2.5. High-Definition Transcranial Direct Current Stimulation

The montages to stimulate the dACC and right posterior insula were defined as in previous studies of our group [52,56] using the ROAST 3.0 toolbox [77,78]. The montages were optimised by modelling 6 mm radius circular electrodes [79] and the indices of volume conduction assigned to different tissues based on a high-resolution T1-weighted MRI image [77]. The optimal solutions resulted in the following electrode placement: for the right insula, 3 anodes on CP4-C6-CP6 and 3 cathodes on FT8-F10-FT10; for the dACC, 3 anodes on Fz-F1-FCz and 3 cathodes on PO9-O9-O10. The two montages were then checked, modelling the current flow location and intensity, considering 2 mm of conductive gel and 9.5 mm radius electrodes. The total delivered intensity was 3 mA, with each anode and cathode acting as source and sink of 1 mA current intensity (with a current density of 0.35 mA/cm^2^ for each electrode). The electrodes, inserted into saline-soaked sponges of the same shape and size, were positioned on participants’ heads using an EEG brain cap (MCS Multi-Cap, Spes medica s.p.a., Genova, Italy) to correctly identify the electrode locations based on the 10–10 system. Three battery-driven neurostimulation devices (Brainstim, EMS s.r.l, Bologna, Italy) synchronised through triggers were used to implement the HD-tDCS.

In anodal sessions, the constant current of 1 mA per active electrode was delivered for 20 min, with 15 s gradually ramping up and down at the beginning and end of stimulation, respectively. In sham sessions, the same ramp-up/down parameters were maintained, but, unknown to participants, the stimulation was delivered only for 30 s at the beginning and again for 30 s at the end of the 20 min protocol. These parameters followed safety guidelines for tDCS applications in humans [80,81,82], and a sham protocol was adopted to ensure participants’ blinding to the stimulation condition [52,56,64,82,83], without inducing an after-effect [84,85].

### 2.6. Procedure

The study implemented a single-blind within-subject design. Following recruitment and screening for eligibility criteria, participants completed the online questionnaires (see above). Then, they came to the laboratory to carry out the baseline DD task, followed by the three HD-tDCS sessions separated by a washout period of at least two days, with the order of anodal insula, anodal dACC, and sham stimulation counterbalanced across participants. Based on previous studies on cortical excitability and cognitive performances, we expected that the 20 min of HD-tDCS elicited an after-effect approximately as long as the stimulation duration [55,56,86,87,88].

**Figure 1:**
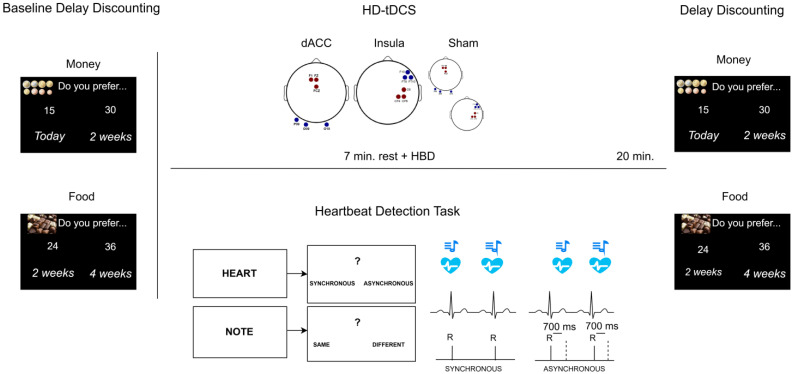
Experimental procedure. Participants completed a baseline delay discounting (DD) task with monetary and food rewards and then three HD-tDCS sessions (anodal insula, anodal dACC, or sham) with counterbalanced order. During the 20 min stimulation, participants rested for 7 min and then completed an online heartbeat detection (HBD) task, including interoceptive and exteroceptive trials. Each trial ended with a forced-choice screen displaying a question mark with the answer options below (Synchronous/Asynchronous in “Heart” trials; Same/Different in “Note” trials). All stimuli were presented either synchronously or asynchronously (700 ms delay) with the heartbeat. Solid lines indicate the onsets of the R-peaks, whereas dashed lines indicate the onset of asynchronous stimuli relative to the R-peaks. Immediately after stimulation, DD tasks with monetary and food rewards were administered, with reward order counterbalanced across participants.

During each session, participants were seated on a comfortable chair in front of an LCD computer screen within a shielded, silent cabin. ECG electrodes were positioned at the left and right collarbones, with the ground positioned under the left rib, then the electrodes for HD-tDCS were placed according to the session montage. Importantly, we aimed to assess the modulatory effect of HD-tDCS on the HBD task, as well as to evaluate the impact on decision-making of dACC–insular circuit activation through the combined action of the interoceptive task and HD-tDCS targeting these two areas. To this end, the HBD task was administered online during the 20 min of HD-tDCS. Participants were asked to stay relaxed for the first 7 min of stimulation; then, the task started and it was completed at approximately the same time as the end of the stimulation. Immediately after the stimulation ended, DD tasks involving monetary and food rewards were administered, with the order of the two reward types balanced across participants (Figure 1). It is worth noting that, although evidence on the greater effect of online vs. offline tDCS protocols on cognitive tasks is contrasting [89,90,91,92], previous findings support state-dependent effects of neurostimulation and suggest circuit-specific modulation of regions related to the cognitive tasks executed online while tDCS is applied [93,94,95]. Thus, we opted for the online protocol to assess the impact on the HBD task of modulating the two cortical hubs of the dACC-insular network, and further explore the possibility that boosting this circuit during the interoceptive task could modulate decision-making following the stimulation.

Participants received course credit and a reimbursement in the form of monetary compensation and a small amount of chocolates/chips, depending on their preference. At the end of each session, participants completed a questionnaire on sensations during the stimulation [96].

### 2.7. Data Analysis

#### 2.7.1. ECG Analyses

The ECG recording was visually inspected for artifacts using a modified Pan and Tompkins algorithm [97,98] implemented in the AcqKnowledge software (version 5.0.10) to detect QRS complexes. Vagally mediated HRV was estimated in the time domain by means of the root mean square of successive RR interval differences (RMSSDs), which were calculated for each trial (Heart/Note~12 s). Data were then analysed in the statistical programming environment R [99]. The RMSSD was obtained by calculating each successive time difference between heartbeats in ms. Then each of the values was squared, and the result was averaged before the square root of the total was obtained. RMSSD values were transformed by the natural logarithm (ln) to account for skewness. This measure is considered the primary time-domain measure used to estimate vagally mediated changes reflected in HRV, which is also employed for ultra-short-term periods of time (10 s, 30 s, and 60 s). RMSSD likely reflects broader vagal regulatory tone during the task and should therefore be interpreted as an index of autonomic context rather than a direct marker of moment-to-moment interoceptive precision [58,100]. To examine the effects of task type and stimulation condition, a two-way repeated-measures ANOVA was conducted on RMSSD values. The within-subject factors were the task type (heart/note) and the tDCS condition (insula, dACC, and sham). Moreover, to explore the relationship between HRV and behavioural measures, we performed Spearman rank correlations between RMSSDs during the sham condition and (1) interoceptive accuracy, (2) the delay discounting index, and (3) the proportion of sooner choices in now trials, computed separately for monetary and food rewards.

#### 2.7.2. Heartbeat Detection Analyses

Analyses on the interoceptive task were carried out introducing the accuracy of HBD as a dichotomous dependent variable in general mixed-effects models [101] fitted using the GLMER function of the lme4 R package (R 4.4.3) [102]. The HD-tDCS condition (factorial, three levels: anodal insula vs. anodal dACC vs. sham), the type of trial (factorial, two levels: heart vs. note), the synchronous condition (factorial, two levels: synchronous vs. asynchronous to individual heartbeat), and their interactions were entered in the full model as fixed factors. Moreover, we added the simple effect of the trial order to account for changes in performance due to learning or fatigue effects and a by-subject random intercept to account for participant specific variability [101]. The inclusion of fixed effects in the final model was tested using a series of likelihood ratio tests (LRTs), and parameters were progressively removed if they did not significantly increase the overall model fit [103]. Furthermore, to assess the effect of practice on task repetition, we conducted a separate analysis considering the accuracy in the HBD as a dependent variable in a repeated-measures ANOVA with factors session order (three levels: first, second, third session) and type of trial (two levels: heart vs. note).

#### 2.7.3. Delay Discounting Analyses

Individual index of delay discounting was estimated by modelling participants’ choices in the DD task through a hyperbolic function [62,76]. Specifically, a one-parameter model of hyperbolic discounting was used to compute the following equation [104]:

SV = 1/(1 + kD)(1)

where SV = the subjective value (expressed as a fraction of the delayed amount), D = the delay (in days), and k = a subject-specific constant of the best fitting delay discounting function, with larger k values reflecting a steeper discounting function and thus a stronger preference for smaller immediate rewards over larger delayed ones.

For each session and reward type in DD tasks, the hyperbolic function was fitted to each participant’s data (i.e., values of sooner options and delayed options, delays, and choices) by estimating a binary logit model via maximum likelihood through a MATLAB-based script (MATLAB R2021a; MathWorks, Inc., Natick, MA, USA) [105]. This method allows us to estimate the individual index of “delay discounting rate” (the subject-specific constant “k, i.e., the decay rate of the subjective value of a reward over time), minimising the difference between the actual individual choices and those predicted by the hyperbolic function. The goodness of model fit was assessed with r2 [106,107]. The DD index was then introduced as a dependent variable in a repeated-measure ANOVA with factors session (four levels: baseline vs. anodal HD-tDCS insula vs. anodal HD-tDCS dACC vs. sham) and reward type (2 levels: Money vs. Food). The effect of session order on DD index was then evaluated through a repeated-measure ANOVA with factors order of session (four levels: first vs. second vs. third vs. fourth) and reward type (2 levels: Money vs. Food).

Moreover, participants’ choices for sooner vs. later options were introduced as dichotomous variables in general mixed effects models, including the session (factorial, four levels: baseline vs. anodal HD-tDCS insula vs. anodal HD-tDCS dACC vs. sham HD-tDCS), the type of reward (factorial, two levels: money vs. food), the type of trial (factorial, two levels: now vs. not now trial) and their interaction as fixed factors in the full model. By-trial and by-subject random effects were also included in the model. A series of likelihood ratio tests (LRTs) was used to test the inclusion of fixed effects in the final model, and parameters were progressively removed if they did not significantly increase the overall model fit [103].

## 3. Results

### 3.1. Blinding Efficacy and Questionnaires

The effectiveness of the blinding procedure was assessed by analysing participants’ responses to tDCS-induced sensations questionnaires [96]. Data support participants blindness to stimulation condition, as only 21.74% of participants were able to correctly identify the stimulation condition (i.e., real or sham) across the three sessions, and no significant differences were found between HD-tDCS to the insula, HD-tDCS to the dACC and sham condition in terms of the proportion of participants reporting to have received real stimulation [60.9%, 91.3%, and 69.6%, respectively; χ^2^(2) = 5.86, *p* = 0.53], and confidence in their judgments [F(2, 36) = 0.70, *p* = 0.51]. Furthermore, no significant differences in subjective sensations were observed across stimulation conditions. Crucially, participants reported low-to-mild sensations across sessions, and nobody experienced any significant adverse effects (see Supplementary Material, Table S1). We also explored the correlations between the behavioural measures of the HBD and DD tasks and the questionnaires on body and interoceptive awareness, emotion regulation, and cognitive flexibility. All correlations, performed by checking Spearman rank coefficients, were non-significant (see Supplementary Material, Table S2).

### 3.2. Heartbeat Detection

Outliers were identified based on Pearson residuals [108] derived from the fitted model as a diagnostic measure. Observations from one subject with residual values exceeding ±3 standard deviations—especially in exteroceptive trials—were excluded from the analyses. In addition, one subject was excluded from the analyses due to technical issues during data acquisition. Thus, 22 participants were included in the final analysis.

The analysis on the full model, including the fixed effects of HD-tDCS condition (anodal insula/anodal dACC/sham), trial type (heart/note) and synchronous condition (synchronous/asynchronous), revealed the significant main effects of trial type (χ^2^(1) = 5471.9, *p* < 0.001) and synchronous condition (χ^2^(1) = 1496, *p* < 0.001), as well as the significant interactions of the HD-tDCS condition x trial type (χ^2^(2) = 39.8, *p* < 0.001), trial type × synchronous condition (χ^2^(1) = 342.8, *p* < 0.001) and the three-way interaction HD-tDCS condition x trial type, x synchronous condition (χ^2^(2) = 52.9, *p* < 0.001). The main effect of the HD-tDCS session was not significant (χ^2^(2) = 4.37, *p* = 0.113). Accuracy was overall higher for the note (mean = 0.91, s.d. = 0.04) than heart trials (mean = 0.48, s.d. = 0.19), and for the synchronous (mean = 0.78, s.d. = 0.15) versus the asynchronous condition (mean = 0.60, s.d. = 0.30). Post hoc analysis with Bonferroni correction for multiple comparisons on the HD-tDCS condition x trial type interaction revealed increased accuracy in both HD-tDCS sessions compared to the sham condition in note trials (i.e., exteroceptive trials; insula vs. sham: *p* < 0.001 and dACC vs. sham: *p* = 0.008) and higher accuracy in dACC vs. insula session (*p* = 0.013). The accuracy was not overall modulated by HD-tDCS in heart trials (*p* > 0.05). However, this effect was further qualified by the three-way interaction showing that, in the asynchronous condition, accuracy was higher during dACC stimulation compared to sham (*p* = 0.001) and compared to insula stimulation (*p* = 0.037) in heart trials, whereas in note trials, accuracy was higher during insula than dACC stimulation (*p* < 0.001). Conversely, when tones were synchronous to participants’ heartbeats, both insula and dACC stimulation decreased accuracy compared to sham in heart trials (*p*= 0.025 and *p* = 0.037, respectively), which is at odds with the note trials, in which both insula and dACC stimulation increased accuracy compared to sham (*p* < 0.001).

Separate analysis on session order revealed a significant trial type by session interaction (*p* = 0.028), suggesting a different pattern of practice effect across sessions for heart and note trials, although post hoc contrasts between sessions were not statistically significant (see Supplementary Material, Tables S3 and S4, and Figure S1).

**Figure 2:**
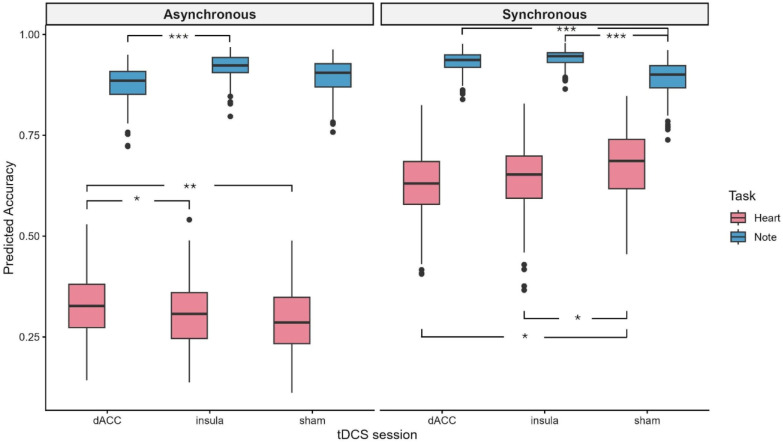
HB detection task results. Boxplots showing predicted accuracy for each trial type (Heart and Note; red and blue, respectively) under asynchronous (**left** panel) and synchronous (**right** panel) conditions across different HD-tDCS sessions (dACC, insula, and sham). The central line indicates the median, boxes represent the interquartile range (IQR), whiskers extend to 1.5 × IQR, and data points falling outside the whiskers denote outliers (not excluded as within 3 standard deviations). Brackets with asterisks indicate significant post hoc pairwise comparisons corrected for multiple testing using the Bonferroni method (* *p* < 0.05, ** *p* < 0.01, and *** *p* < 0.001).

To summarise, both insula and dACC stimulation interfered with participants’ capacity to recognise synchronous heartbeat, whereas dACC stimulation improved identification of asynchronous beats (see Figure 2). On the other hand, accuracy in exteroceptive trials improved during insula and dACC stimulation.

### 3.3. HRV Analyses

The repeated-measures ANOVA on RMSSD values to assess HRV revealed no significant effects of the task type (heart/note; F(1, 16) = 0.060, *p* = 0.81), HD-tDCS condition (insula, dACC, sham; F(1, 32) = 0.29, *p* = 0.75), and interaction task type x HD-tDCS condition (F(1, 32) = 0.078, *p* = 0.93).

HRV did not significantly correlate with interoceptive accuracy (ρ = 0.29; *p* = 0.25). For the money condition, no significant correlations were found between HRV (RMSSD) and any of the behavioural measures: delay discounting index (ρ = –0.03, *p* = 0.32) and proportion of immediate (“now”) choices (ρ = 0.32, *p* = 0.21). Similarly, for the food condition, HRV was not significantly correlated with any behavioural measure: delay discounting index (ρ = 0.01, *p* = 0.96) and proportion of immediate choices (ρ = 0.04, *p* = 0.87).

### 3.4. Delay Discounting 

**Figure 3:**
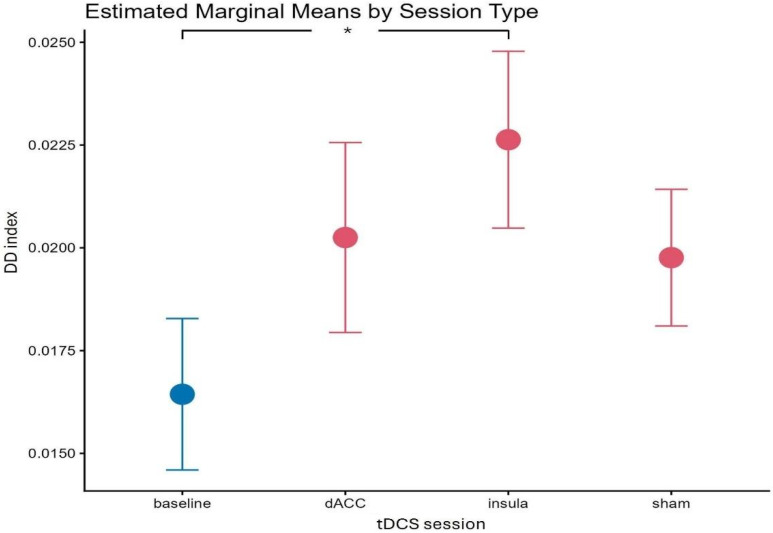
DD results from repeated-measures ANOVA on a hyperbolic model. The line plot shows the estimated marginal means of the hyperbolic delay discounting index across tDCS sessions (baseline, dACC, insula, and sham; baseline in blue and stimulation conditions in red). Error bars represent ±1 standard error. Brackets with asterisks indicate significant post hoc pairwise comparisons between session types, corrected for multiple comparisons using the Bonferroni method (* *p* < 0.05).

The repeated-measures ANOVA on the hyperbolic estimation of delay discounting index revealed a significant main effect of reward type (F(1, 23) = 8.89, *p* = 0.007), indicating steeper discounting for food (mean = 0.023, sd = 0.01) compared to monetary rewards (mean = 0.016, sd = 0.009) and a significant main effect of the HD-tDCS session (F(1, 23) = 3.54, *p* = 0.019). Post hoc Bonferroni-corrected comparisons showed that discount rates were significantly increased after the insula stimulation (mean = 0.023, sd = 0.01) compared to baseline (mean = 0.016, sd = 0.009; *p* = 0.026; see Figure 3). The reward type × HD-tDCS session interaction effect was not significant (F(1, 23) = 1.91, *p* = 0.14).

The analysis on session order showed a significant main effect of task repetition (*p* = 0.032), but post hoc contrasts between sessions did not support significant differences between them related to the repetition of the task, notably including contrast between the first baseline vs. second, third, or fourth experimental session (see Supplementary Material, Tables S5 and S6 and Figure S2).

A general mixed-effect model was performed to further examine the influence of stimulation and reward type on participants’ choice for sooner vs. later options. The best fitting model included the main effects of HD-tDCS session (χ^2^(3) = 47.6, *p* < 0.001), reward type (χ^2^(1) = 170.1, *p* < 0.001), and trial type (χ^2^(1) = 4.38, *p* = 0.037), as well as a significant HD-tDCS session × reward type interaction (χ^2^(3) = 7.99, *p* = 0.046). Model comparison based on the Akaike Information Criterion (AIC) and LRTs indicated that the most parsimonious model, excluding the interaction with trial type, provided the best fit to the data (AIC_full = 17,291 vs. AIC_reduced = 17,282), without a significant loss of explanatory power (*p* = 0.67).

Participants’ choices for sooner options were significantly higher in now trials (mean = 0.692, s.d. = 0.13) than not now trials (mean = 0.676, s.d. = 0.13), and in the DD task with food reward (mean = 0.73, s.d. = 0.12) compared to money rewards (mean = 0.64, s.d. = 0.13). Furthermore, participants’ choices for sooner options were significantly higher during HD-tDCS sessions compared to the baseline (all ps < 0.05); dACC mean = 0.69, s.d. = 0.13; insula mean = 0.7; s.d. = 0.13; sham mean = 0.71, s.d. = 0.13; baseline mean = 0.64, s.d. = 0.13).

**Figure 4:**
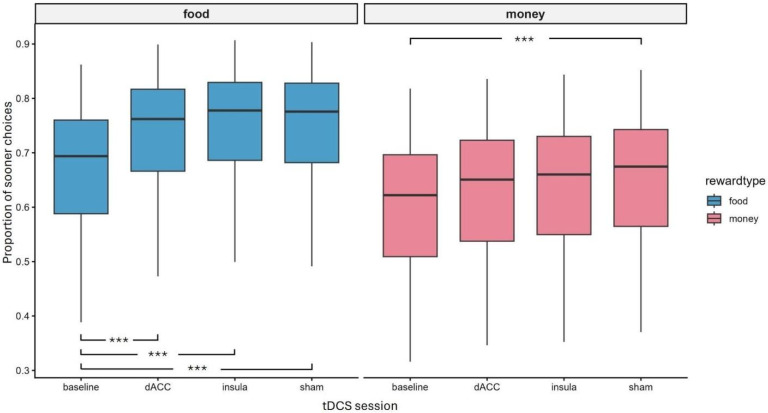
DD task results. Boxplots showing the proportion of sooner choices for each reward type (food and money; blue and red, respectively) across the HD-tDCS sessions (baseline, dACC, insula, and sham). The central line indicates the median, boxes represent the interquartile range (IQR), and whiskers extend to 1.5 × IQR. Brackets with asterisks indicate significant post hoc pairwise comparisons corrected for multiple testing using the Bonferroni method (*** *p* < 0.001).

Looking at the two-way interaction (Figure 4), post hoc analyses showed significant differences between baseline and all stimulation sessions when food reward was presented (baseline vs. dACC, insula, and sham; all *p* < 0.001). Instead, in the DD task with a monetary reward, only the comparison between baseline and sham reached significance (*p* = 0.01).

## 4. Discussion

The present study aimed to evaluate the effectiveness of a mixed online–offline HD-tDCS protocol targeting the right insula and dACC to modulate interoceptive accuracy and impulsivity in decision-making across different reward types (i.e., food and money). In particular, we expected (1) a modulation of the HBD task during insula stimulation and (2) a modulation of impulsivity in DD tasks performed following active HD-tDCS sessions paired with the interoceptive task. We further hypothesised (3) to detect a greater modulation following real HD-tDCS for food reward decision-making and (4) associations between HRV and both interoceptive accuracy and impulsivity.

Consistently with our first hypothesis, we found significant effects of HD-tDCS to the right insula on the accuracy of the HBD task. Interestingly, dACC stimulation also affected HBD performances, and these modulations differed according to whether the trials presented tones that were synchronous or asynchronous with the participants’ heartbeat and whether they required interoceptive or exteroceptive processing. Specifically, in trials synchronised with participants’ heartbeat, the HD-tDCS to both insula and dACC impaired interoceptive accuracy compared to sham (i.e., reduced accuracy in recognising synchronous heart trials), whereas exteroceptive capacity improved compared to the sham session (i.e., accuracy in tone discrimination increased). Insula stimulation also increased exteroceptive accuracy in asynchronous trials compared to dACC. Moreover, dACC increased accuracy in the interoceptive task with asynchronous trials compared to sham and insula conditions (i.e., improving the detection of tones delayed with respect to participants’ heartbeat).

Our results support and extend previous findings reporting a reduced capacity in detecting interoceptive signals following anodal tDCS of the right and left insula [47], which suggested an interferential effect of the stimulation possibly related to an alteration of the intra-insula signal (i.e., the functional connectivity between posterior and anterior insula; [109]). Concurrently, the improvement in exteroceptive accuracy during HD-tDCS indicates that the insula’s attentional monitoring role within the salience network was not only maintained but further strengthened by the stimulation [110]. It is important to note that our stimulation targeted the right posterior insular cortex, which is regarded as a critical hub for interoceptive processing and for mediating decision-making behaviour via the integration of interoceptive signals [111,112]. This aligns with the model of interoceptive predictive coding, or interoceptive inference [113], which posits a posterior-to-anterior gradient in the insula, where the posterior subregion is primarily involved in mapping interoceptive signals (i.e., visceral interoception), whereas the anterior part contributes to higher-order re-representation and integration of interoceptive and exteroceptive signals [109,114]. Support for this hierarchical organisation also comes from findings showing altered reward valuation and dysfunctions of the right posterior insula in drug-addicted individuals [115]. In particular, within the predictive coding framework, interoception arises from hierarchical generative models, which are continuously updated by the brain’s attempts to minimise the prediction error (i.e., the discrepancy) between the sensory input and the emerging models [113]. The insula is considered a crucial comparator for predictive coding, with the posterior–anterior interactions likely supporting the integration of physiological signal and visceral representation. This integration is further modulated by mechanisms of precision weighting, possibly related to post-synaptic signal, which determine the extent to which prediction errors are resolved [116]. Our findings support the role of the insula as a comparator for predictive coding, although interpretations of the HD-tDCS effects in terms of synaptic connectivity or intra-insular communication can only be speculative.

Concerning dACC, our results align with previous studies suggesting its role as a conflict resolution hub continuously integrating internal and external information [33,117,118]. This is also convergent with recent evidence proposing the dACC as a visceromotor effector brain region contributing to interoceptive coding by regulating bodily arousal in relation to decision-making [119,120]. In particular, neurons decoding heart rate and the current state of bodily arousal have been described in macaques [120]. In line with this evidence, our data support the role of dACC in heartbeat processing.

Taken together, the present results support the combined role of the insula and dACC in interoceptive representations. The HD-tDCS may have disrupted interoceptive processing of the insula by increasing noise within the posterior–anterior insula communication stream, or reducing the precision weighting required for prediction error updates. On the other hand, dACC stimulation has modulated the encoding of individual’s heartrate, impairing the ability to recognise synchronous heartbeat while improving accuracy in asynchronous trials, possibly for the enhancement of cognitive control when integrating internal and external cues.

Concerning decision-making behaviour, assessed by using the delay discounting task with food and monetary rewards, we found a more impulsive behaviour when participants faced food decisions compared to monetary decisions, as indexed by a greater discounting rate and a higher proportion of sooner choices in food blocks. This is consistent with previous behavioural and neuroimaging evidence showing domain specificity of temporal discounting, with higher impulsivity for food decisions, and partially distinct circuits at the brain level for primary and secondary reward stimuli [24,121].

In line with our second hypothesis, the discounting index overall increased following HD-tDCS to the insula compared to baseline, supporting the role of this region in reward-based decision-making [122]. It is important to highlight that HD-tDCS was applied during the HBD task. Significant differences were found between insula and baseline decision-making, whereas discounting rate following dACC stimulation and the sham condition was not significantly modulated. Following our previous interpretation on HD-tDCS perturbating the insular interoceptive processing, this result further suggests a disruption in the posterior-to-anterior neural signal crucial for the integration of interoceptive representation in driving decision-making [111]. In other words, the HD-tDCS to the insula appeared to interfere with the interoceptive processing, leading to higher impulsivity in evaluative decision-making. In line with this view, recent evidence indicates that reduced insular activity can heighten delay discounting rates in individuals with obesity, thereby contributing to greater impulsivity in reward-based decisions [123]. It is also worth noting that a previous study from our group targeting the insula with the same HD-tDCS montage reported the absence of effect on risk and loss aversion and on conflict effect in a Flanker task [52]. In addition to differences in decision-making tasks, the study applied the stimulation offline at rest. Thus, the present significant finding supports the state-dependent effect of tDCS, which may induce circuit-specific modulation when applied concurrently to task execution [93,94,95].

Our third hypothesis was to identify greater modulation for real HD-tDCS, particularly for food rewards, as interaction with the interoceptive network was expected to have a greater impact on food-related decision-making [17]. This was only partially supported by data. Indeed, insula stimulation increased discounting rate compared to baseline but not compared to the sham session, and overall, across monetary and food reward. On the other hand, analysis on proportion of impulsive choices (i.e., selection of the sooner option) revealed that the effects of HD-tDCS sessions varied as a function of reward type. Specifically, impulsive choices towards food increased following sessions with HD-tDCS and HBD compared to the baseline irrespectively of stimulation condition. In monetary conditions, a significant increase in impulsive choices resulted in the sham condition compared to the baseline, but not following the insula and dACC stimulations. Thus, it appears that engagement in HBD increased impulsivity for food reward, likely heightening the individual salience of bodily states, and real or sham HD-tDCS did not modulate this effect. Instead, monetary impulsive choices increased following HBD with sham stimulation compared to baseline, but not following dACC and insula stimulation. One possible interpretation is that HD-tDCS, by interacting with the interoceptive circuit, mitigated the salience effect of bodily state on impulsive behaviour when this was directed towards stimuli less associated with the perception of internal states. However, caution is needed in this interpretation, and future studies could directly test the interplay between the interoceptive circuit and decision-making with a multimodal approach. For instance, simultaneous tDCS-fMRI and tACS-fMRI studies can be designed to assess the effects of neuromodulation on functionally connected brain regions, both in task-based and resting state conditions [124,125].

Finally, our results showed no significant correlations between HRV with either interoceptive accuracy or impulsivity, at odds with our fourth hypothesis. Previous studies reported inconsistent results on the association between interoceptive processing and HRV [14,59,60,61]. Moreover, the intrinsically high individual variability of HRV as a physiological index [126], and the dimensional model of interoception, encompassing different facets of interoceptive processing (i.e., interoceptive accuracy, sensitivity, and awareness; [127,128,129]) may suggest that different cardiac measures could be related to distinct dimensions of interoceptive processing [130]. Future studies implementing interoceptive and decision-making paradigms, even in combination with HD-tDCS, may therefore benefit from adopting alternative metrics, such as heart-evoked potentials (HEPs), which constitute a promising neural marker for investigating the effects of neuromodulation on interoception [131,132].

Overall, our results need to be interpreted in light of some limitations. First, we modelled HD-tDCS montages using a high-resolution standard head model (i.e., the New York Head, [77]) rather than individual MRI from our participants. This approach has known methodological constraints [133], as it does not account for individual anatomical differences [134,135] and may lead to unintended stimulation of non-targeted brain regions [136]. More generally and beyond modelling predictions, the role of individual differences in tDCS responsivity (i.e., morphological characteristics of the brain and state-dependency of targeted cortical regions) must also be taken into account when evaluating modulatory effects [91]. The within-subject design has been selected to improve control on inter-individual variability; however, we must acknowledge that the limited sample size and the design involving interactions of multiple experimental conditions did not allow for further investigation into the potential sources of variability in task performance and HD-tDSC neuromodulatory effects. Another limitation is the absence of an additional control condition or control stimulation site in the experimental design. For instance, a sham stimulation condition without the interoceptive task would have allowed a direct comparison with the effects at the baseline. However, the decision not to include an additional session was based on the objective of limiting as much as possible the learning effect of the task. Notably, analyses of session order revealed that performance was affected by practice. However, the counterbalancing of tDCS sessions, coupled with the lack of significant direct comparisons between sessions based on their order, supports the causal role of HD-tDCS in task modulation. A further potential limitation is inherent in the task of HBD. Its reliability has been proven in different studies, although some methodological aspects such as the delay of tones for asynchronous conditions, the number and type of trials have been debated [57,137,138]. Moreover, the accuracy tends to be low and some participants report guessing in interoceptive trials [29]. In our study we did not collect confidence scores, thus we cannot drive conclusion on the role of interoceptive awareness nor on its modulation by HD-tDCS. On the other hand, our main objective was to engage the insular-dACC circuit during HD-tDCS sessions in order to test the neuromodulatory effect online. Indeed, the HBD task has been consistently reported to activate these cortical areas [29] and our results causally support their role in task performances.

Although the present study was conducted in a non-clinical sample, these findings may have potential translational relevance, informing the development of HD-tDCS protocols in combination with interoceptive measures. Interoception and impulsivity are increasingly conceptualised as transdiagnostic categories across several psychiatric conditions [139,140], with particular emphasis on EDs and addiction, in which an altered homeostatic regulation has been proposed to play a central role [16,141]. Indeed, impairments in the communication among components of the bodily feedback system (i.e., signal, perception and appraisal) have been described in individuals with obesity, as well as in patients with addiction, resulting in altered reward evaluation and dysregulated interoceptive processing [17,142]. Similar alterations in the balance between interoceptive mechanisms and reward-based behaviour have been reported in patients with Parkinson’s disease [143,144]. In this context, our results appear promising in modulating these mechanisms by inducing circuit-specific effects in the insular–dACC network. However, further studies with clinical populations are needed to directly test the translational implications of HD-tDCS protocols targeting interoceptive processing and impulsive decision-making.

## 5. Conclusions

This study contributes to the growing literature on the roles of the insula and dACC in regulating the complex interplay between impulsive decision-making, reward valuation—especially with food stimuli—and interoceptive processing. To the best of our knowledge, no previous studies have employed model-based HD-tDCS in a mixed online–offline protocol involving both interoceptive and reward-based decision-making tasks with food and monetary rewards. Our findings support the crucial role of the right insula and the dACC in interoceptive representations. They also suggest that insular cortex modulation impacts the integration of this processing into evaluative decision-making. However, the neuromodulatory effects of HD-tDCS on impulsive choices warrant cautious interpretation, as they varied as a function of reward type. Notably, higher impulsivity was observed for food compared to monetary rewards, highlighting the importance of stimulus type when designing and evaluating interventions targeting impulsive behaviour and dysregulated eating.

## Data Availability

Data available on https://osf.io/zck93/metadata/osf (accessed date on 9 July 2025).

## Supplementary Materials

The following supporting information can be downloaded at: https://www.mdpi.com/article/10.3390/biomedicines14030519/s1, Table S1: Mean values and standard deviations of tDCS-induced sensations across all stimulation sessions (HD-tDCS targeting dACC. HD-tDCS targeting insula. Sham). For each cell. it is also reported the percentage (%) of participants reporting a value > 2 (i.e. considerable or strong sensation); Table S2: Correlations between the behavioural measures of the HBD and DD tasks and the questionnaires on body and interoceptive awareness. emotion regulation and cognitive flexibility. All correlations were performed by checking Spearman rank coefficients; Table S3: Results from the analysis on HBD task considering the session order as an independent variable. An ANOVA with trial type (heart vs. note) and session order (1–2 and 3rd session) as independent variables was performed on the accuracy of the HBD task.; Table S4: Results of post hoc analysis on the interaction trial type by session order (X1= 1st session, X2 = 2nd session, X3 = 3rd session). Paired contrasts between each session were conducted for the heart and note trials; Table S5: Results from the analysis on DD index considering the session order as an independent variable. An ANOVA with reward type (money x food) and session order (1–2–3 and 4th session) as independent variables was performed on the individual discounting index modelled from DD; Table S6: Results of post hoc analysis on the main effect of session order (X1= baseline, X2 = 1st session, X3 = 2nd session, X4 = 3rd session). Paired contrasts between each session were conducted. Figure S1: Line plot of the estimated marginal means from the Anova on accuracy in HBD task. The accuracy across sessions (X1= 1st session, X2 = 2nd session, X3 = 3rd session) in heart and note trials is depicted. Error bars represent ±1 standard error. Results showed a significant trial type (note/heart) by session interaction in the HBD task suggesting a different impact of task repetition on note and heartbeat detection, however post hoc contrasts between sessions were not significant for either types of trial; Figure S2: Line plot of the estimated marginal means from the Anova on the hyperbolic delay discounting index across the sessions (X1= baseline, X2 = 1st session, X3 = 2nd session, X4 = 3rd session). Error bars represent ±1 standard error. The main effect of task repetition was significant in the analysis on DD index, but no significant results emerged from post hoc contrasts between sessions.

## Abbreviations

The following abbreviations are used in this manuscript:

HRV	Heart Rate Variability
ANS	Autonomic Nervous System
EDs	Eating Disorders
DD	Delay Discounting
BMI	Body Mass Index
dACC	Dorsal Anterior Cingulate Cortex
TMS	Transcranial Magnetic Stimulation
tDCS	Transcranial Direct Current Stimulation
HD-tDCS	High-Definition tDCS
HBD	Heartbeat Detection
ECG	Electrocardiogram
EHI	Edinburgh Handedness Inventory
BPQ	Body Perception Questionnaire
DEBQ	Dutch Eating Behaviour Questionnaire
DERS-SF	Difficulties in Emotion Regulation Scale-Short Form
MAIA	Multidimensional Assessment of Interoceptive Awareness
RMSSDs	Root Mean Square of Successive RR interval Differences
LRTs	Likelihood Ratio Tests
HEPs	Heart-Evoked Potentials
