# Supplementary material for: Modulating the Interplay Between Impulsivity and Interoception Through HD-tDCS to the Right Insula and Anterior Cingulate Cortex
Source: Biomedicines. 2026 Feb 26;14(3):519. doi: 10.3390/biomedicines14030519 (PMC13023666; doi:10.3390/biomedicines14030519)

Supplementary material

Table S1. Mean values and standard deviations of tDCS-induced sensations across all stimulation sessions (HD-tDCS targeting dACC. HD-tDCS targeting insula. Sham). For each cell. it is also reported the percentage (%) of participants reporting a value > 2 (i.e. considerable or strong sensation).

Session	Itching	Pain	Burning	Heat	Tingling	Metallic taste	Fatigue
HD-dACC (Anodal)	1.17 (1.11) 17.39%	0.87 (1.01) 13.04%	1.30 (1.15) 26.09%	0.48 (0.67) 0%	1.26 (1.25) 17.39%	0.17 (0.83) 4.35%	0.44 (0.95) 8.70%
HD-insula (Anodal)	1.00 (0.85) 8.70%	0.57 (0.73) 0%	0.83 (1.03) 4.35%	0.35 (0.78) 4.35%	1.09 (1.08) 13.04%	0.17 (0.83) 4.35%	0.44 (0.79) 4.35%
Sham	1.13 (0.92) 8.70%	0.65 (0.83) 4.35%	1.09 (1.28) 17.39%	0.61 (0.99) 8.70%	0.83 (1.03) 13.4%	0.13 (0.63) 4.35%	0.22 (0.52) 0%
Across session statistics	F(2.44)=0.40. p=.68	F(2.44)=1.37. p=.27	F(2.44)=1.93. p=.16	F(2.44)=1.63. p=.21	F(2.44)=1.59. p=.22	F(2.44)=1.00. p=.38	F(2.44)=3.02. p=.06

Table S2. Correlations between the behavioural measures of the HBD and DD tasks and the questionnaires on body and interoceptive awareness. emotion regulation and cognitive flexibility. All correlations were performed by checking Spearman rank coefficients.

Variable	**DD_index**				**DD_index**				**Accuracy_HBD**	
Trial type	**Money**		**Money**		**Food**		**Food**		**Money-Food**	
Condition	Baseline		Sham		Baseline		Sham		Sham	
	ρ	*p.value*	ρ	*p.value*	ρ	*p.value*	ρ	*p.value*	ρ	*p.value*
BPQ_1	0.007	0.97	0.158	0.534	0.297	0.195	0.341	0.091	0.194	0.321
BPQ_2	-0.024	0.924	0.182	0.254	-0.018	0.487	0.332	0.423	0.104	0.253
BPQ_3	-0.128	0.495	0.177	0.505	0.213	0.481	0.481	0.035	0.323	0.076
MAIA_1	0.094	0.849	0.208	0.362	0.113	0.953	0.089	0.896	0.314	0.308
MAIA_2	0.04	0.992	0.018	0.798	0.387	0.164	0.284	0.327	0.19	0.283
MAIA_3	-0.101	0.518	0.218	0.383	-0.33	0.418	-0.021	0.775	0.135	0.489
MAIA_4	0.478	0.117	0.144	0.927	0.131	0.455	-0.23	0.724	0.002	0.487
MAIA_5	0.024	0.845	-0.004	0.799	-0.045	0.622	-0.203	0.903	0.086	0.819
MAIA_6	0.383	0.103	-0.084	0.971	-0.042	0.666	-0.314	0.251	-0.377	0.139
MAIA_7	0.114	0.369	-0.002	0.987	-0.144	0.98	-0.146	0.632	-0.289	0.232
MAIA_8	0.109	0.391	-0.003	0.979	0.01	0.846	0.075	0.797	-0.036	0.902
DERS_1	0.099	0.619	0.066	0.968	0.25	0.531	0.131	0.936	-0.129	0.641
DERS_2	0.27	0.447	0.31	0.125	0.269	0.289	0.476	0.115	-0.016	0.68
DERS_3	0.381	0.094	0.23	0.317	0.154	0.907	0.172	0.773	-0.07	0.872
DERS_4	0.374	0.146	0.315	0.321	0.111	0.965	0.159	0.765	-0.057	0.792
DERS_5	0.252	0.342	0.149	0.66	0.138	0.843	0.142	0.817	-0.017	0.946
DERS_6	0.188	0.465	0.049	0.86	0.052	0.918	0.018	0.989	-0.002	0.982
DERS_7	0.361	0.192	0.263	0.41	0.182	0.725	0.245	0.608	-0.065	0.948
DEBQ_1	0.113	0.362	-0.059	0.921	0.297	0.304	0.061	0.835	0.042	0.884
DEBQ_2	0.303	0.198	0.166	0.541	0.163	0.555	-0.071	0.86	-0.053	0.549
DEBQ_3	-0.017	0.949	-0.365	0.095	0.213	0.569	-0.119	0.291	-0.129	0.786
Psy.flex	-0.093	0.926	-0.213	0.71	-0.068	0.887	-0.398	0.25	-0.077	0.561

Table S3. Results from the analysis on HBD task considering the session order as an independent variable. An ANOVA with trial type (heart vs note) and session order (1 - 2 and 3rd session) as independent variables was performed on the accuracy of the HBD task.

HBD task accuracy				
	Sum of squares	Df	F-value	Sig
Trial type	7.129	24	321.9541	2.07e-15 ***
Session order	0.051	48	2.2804	0.11322
Trial type* Session order	0.043	48	3.8701	0.02765 *

Greenhouse-Geisser and Huynh-Feldt Corrections for departure from Sphericity was applied
'***' p<.0001, '**' p = .001, '*' p = .01

Table S4. Results of post hoc analysis on the interaction trial type by session order (X1= 1st session, X2 = 2nd session, X3 = 3rd session). Paired contrasts between each session were conducted for the heart and note trials.

**Task = Heart**	Estimate	SE	df	Sig
X1 - X2	-0.004	0.0297	24	0.9901
X1 - X3	-0.070	0.0294	24	0.0641
X2 - X3	-0.066	0.0298	24	0.0892

**Task = Note**	Estimate	SE	df	Sig
X1 - X2	-0.036	0.0244	24	0.3205
X1 - X3	-0.020	0.0243	24	0.6931
X2 - X3	0.016	0.0131	24	0.4546

Figure S1. Line plot of the estimated marginal means from the Anova on accuracy in HBD task. The accuracy across sessions (X1= 1st session, X2 = 2nd session, X3 = 3rd session) in heart and note trials is depicted. Error bars represent ±1 standard error. Results showed a significant trial type (note/heart) by session interaction in the HBD task suggesting a different impact of task repetition on note and heartbeat detection, however post hoc contrasts between sessions were not significant for either types of trial.

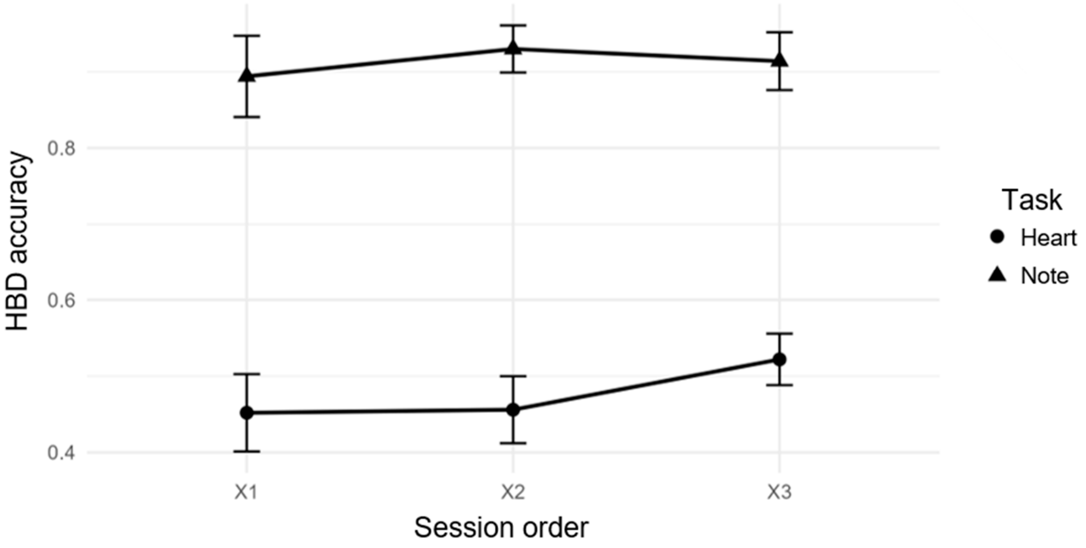

Table S5. Results from the analysis on DD index considering the session order as an independent variable. An ANOVA with reward type (money x food) and session order (1 - 2 - 3 and 4th session) as independent variables was performed on the individual discounting index modelled from DD.

**DD hyperbolic model**				
	Sum of squares	Df	F-value	Sig
Reward type	0.002337	21	8.8960	0.007098 **
Session order	0.000819	63	3.1150	0.032342 *
Reward type *Session order	0.000077	63	0.5247	0.666874

Table S6. Results of post hoc analysis on the main effect of session order (X1= baseline, X2 = 1st session, X3 = 2nd session, X4 = 3rd session). Paired contrasts between each session were conducted.

	Estimate	SE	df	Sig
X1 - X2	-0.00432	0.00175	21	0.1358
X1 - X3	-0.00313	0.00167	21	0.4479
X1 - X4	-0.00588	0.00219	21	0.0828
X2 - X3	0.00119	0.00238	21	1.0000
X2 - X4	-0.00157	0.00210	21	1.0000
X3 - X4	-0.00276	0.00178	21	0.8233

Figure S2. Line plot of the estimated marginal means from the Anova on the hyperbolic delay discounting index across the sessions (X1= baseline, X2 = 1st session, X3 = 2nd session, X4 = 3rd session). Error bars represent ±1 standard error. The main effect of task repetition was significant in the analysis on DD index, but no significant results emerged from post hoc contrasts between sessions.

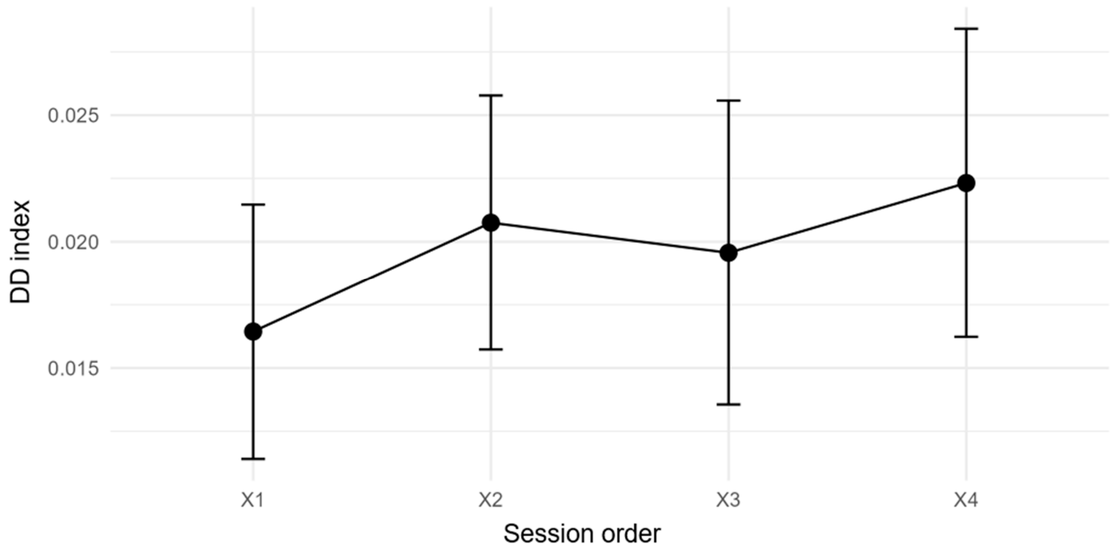

Supplement: Supplementary file 1 [file biomedicines-14-00519-s001.zip › biomedicines-4121442-supplementary.pdf]
